# Supplementary material for: Splice-Junction-Based Mapping of Alternative Isoforms in the Human Proteome
Source: Cell Rep. Author manuscript; Available in PMC 2020 Jan 15. (PMC6961840; doi:10.1016/j.celrep.2019.11.026)
Supplement: 2 [file NIHMS1546469-supplement-2.zip › DF1/PXD006675/Aorta/Aorta_5_SYPL1_ETSLHSPSNTSAPHSQGGIPPPTGL.pdf]

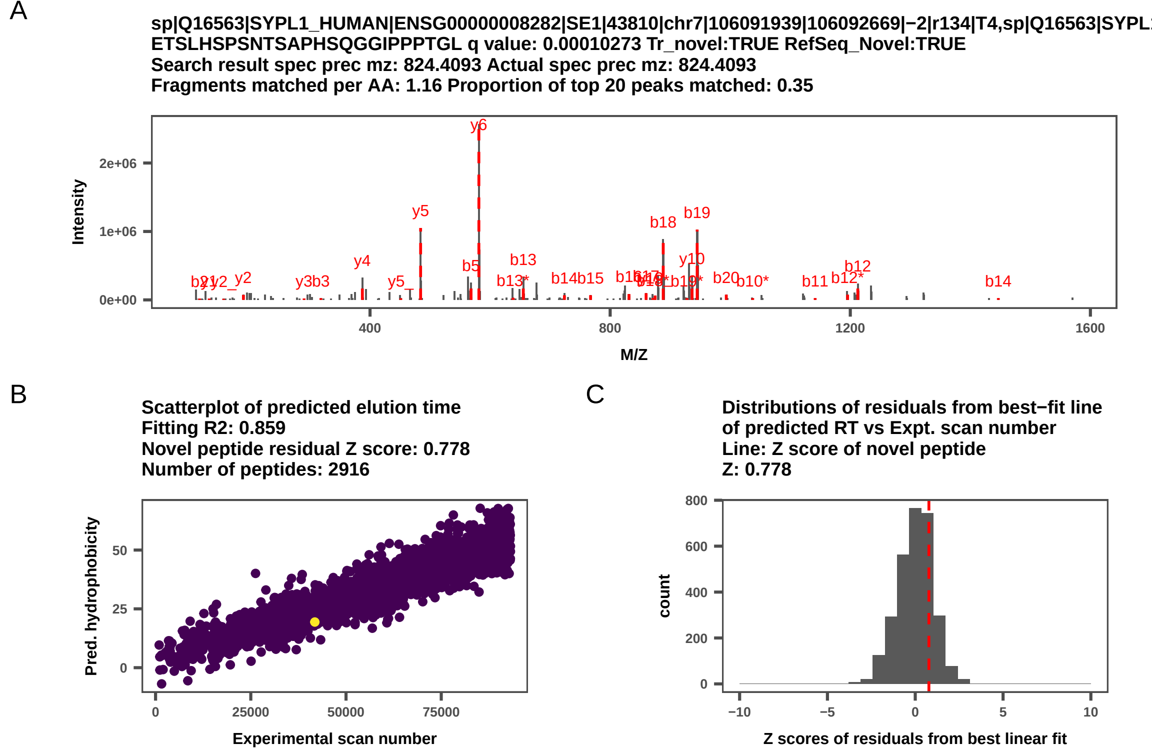
A
sp|Q16563|SYPL1_HUMAN|ENSG00000008282|SE1|43810|chr7|106091939|106092669|−2|r134|T4,sp|Q16563|SYPL
ETSLHSPSNTSAPHSQGGIPPPTGL q value: 0.00010273 Tr_novel:TRUE RefSeq_Novel:TRUE
Search result spec prec mz: 824.4093 Actual spec prec mz: 824.4093
Fragments matched per AA: 1.16 Proportion of top 20 peaks matched: 0.35
Intensity
2e+06
1e+06
0e+00
y6
y5
b18
b19
y10
y4
b5
b13
b13*
b14
b15
b20
b10*
b11
b12
b12*
b14
y3
b3
y2
y5_
400
800
1200
1600
M/Z
B
Scatterplot of predicted elution time
Fitting R2: 0.859
Novel peptide residual Z score: 0.778
Number of peptides: 2916
Pred. hydrophobicity
50
25
0
0
25000
50000
75000
Experimental scan number
C
Distributions of residuals from best−fit line
of predicted RT vs Expt. scan number
Line: Z score of novel peptide
Z: 0.778
count
800
600
400
200
0
−10
−5
0
5
10
Z scores of residuals from best linear fit
